# Relationship between anticipatory, consummatory anhedonia and disorganization in schizotypy

**DOI:** 10.1186/s12888-014-0211-1

**Published:** 2014-08-01

**Authors:** Gwenolé Loas, Annie Verrier, Jean Louis Monestes

**Affiliations:** University Department of Psychiatry, Hôpital Pinel, CHU d’Amiens, EA 4559 Amiens, France; University of Picardie, Amiens, France; University Department of Psychiatry, Hôpital Erasme, Université libre de Bruxelles (ULB), 808 route de Lennick, 1070 Bruxelles, Belgium

**Keywords:** Anticipatory anhedonia, Consummatory anhedonia, Disorganization, Ambivalence, Schizotypy

## Abstract

**Background:**

The disorganized and negative dimensions of schizotypy are characterized by cognitive disorganization and anhedonia, respectively. The aim of the study was to investigate the relationships between these two dimensions of schizotypy by taking into account ambivalence and the distinction between consummatory and anticipatory anhedonia.

**Methods:**

Dimensional analysis and categorical analysis were performed on two different samples (N = 400 and 399) of university students. Self-reported scales were used to measure cognitive disorganization, anticipatory and consummatory anhedonia, and ambivalence. Dimensional analysis using confirmatory factorial analysis examined various models of disorganized and negative schizotypy and categorical analysis compared the scores on anticipatory, consummatory anhedonia and ambivalence scales between various groups of subjects presenting either disorganized schizotypy or negative schizotypy or free of schizotypy.

**Results:**

The disorganized dimension of schizotypy was characterized by schizotypal ambivalence and anticipatory anhedonia, while the negative dimension of schizotypy was characterized by anticipatory and consummatory anhedonia.

**Conclusion:**

The results suggested firstly that ambivalence was not specific of disorganized schizotypy and secondly that anticipatory anhedonia was not specific of negative schizotypy.

## Background

Schizophrenia is a heterogeneous disorder characterized by cognitive and emotional deficits.

Exploratory or confirmatory factor analyses on various rating scales exploring schizophrenia symptoms have commonly reported at least three symptom dimensions: (1) positive (e.g., hallucinations, delusions); (2) negative (e.g., affective flattening, apathy); (3) disorganized (e.g., formal thought disorders, inappropriate affect [1].

Schizotypy is a personality organization characterized by traits that are similar to symptoms found in schizophrenia but often in a less severe form and schizotypy is thought to reflect an underlying vulnerability for schizophrenia [2,3]. Schizotypy is a multidimensional construct and the number of dimensions is highly dependent on the rating scales used to assess schizotypal traits.

Bentall et al [4] underlined that schizotypy scales were developed from three perspectives: symptom-oriented, syndrome-oriented and personality-oriented. In the first approach, individual symptoms were used in the construction of the scales. For example, the Wisconsin schizotypy scales have been developed by the Chapman group using the Meehl manual for schizotypal signs and symptoms.

The Wisconsin schizotypy scales are: physical (PAS) and social anhedonia scales (SanS) [5], perceptual aberration scale (PerAb) [6], magical ideation scale (MIS) [7], impulsive nonconformity scale (INC) [8], cognitive slippage Scale (CSS) [9], social fear scale (SF) [10] and schizotypal ambivalence scale (SAS) [11].

In the second approach, syndrome-based, schizotypy scales were based on criteria of personality disorders. For example, the Schizotypal Personality Questionnaire (SPQ) [12] was developed to rate the nine DSM criteria for schizotypal personality disorder: social anxiety, no close friends, constricted affect, paranoia, magical thinking, unusual perceptual experiences, ideas of reference, odd speech, and odd behaviour.

In the third approach using the domain of personality and individual differences several rating scales have been proposed as the Psychoticism subscale [13] or the Schizophrenia Proneness Scale of the MMPI-2 [14].

The number of dimensions of schizotypy has been examined by factor studies and a review of the studies [15] revealed the constant presence of positive and negative dimensions. For example, Kwapil et al [16] using confirmatory factorial analyses (CFA) on the Wisconsin schizotypy scales (PAS, SanS, PerAb, MIS) in 6137 non-clinical young adults found that the two-factor solution with positive and negative dimensions had the best goodness of fit indices.

When the SPQ was used instead the Wisconsin schizotypy scales three or four structures were found including always a positive (hallucinations, delusions), negative (social anhedonia, anxiety) and disorganization dimensions [17,18].

It is interesting to note that the factorial analyses on the Wisconsin scales did not report a disorganization dimension even when the CSS, measuring cognitive slippage, was included in the design of the study. Only two studies have included the CSS in exploratory factorial analyses of the Wisconsin schizotypy scales.

In 266 undergraduate students Kelley and Coursey [19], using all the Wisconsin schizotypy scales except the Social anhedonia scale, found a two-factor solution with a first factor representing a general schizotypy dimension and a second factor representing an anhedonia dimension (rated by the Physical anhedonia scale). Yon et al in 399 [20] university students using the PAS, SanS, PerAb, MIS, CSS and SAS found a two-factor solution with a first factor representing the positive component of schizotypy (PerAb, MIS, CSS, SAS, SanS) and a second factor representing the negative component of the schizotypy (SanS, PAS). In this study the social anhedonia scale loaded on the two factors, the Physical anhedonia scale loaded only on the negative factor and the Cognitive Slippage Scale loaded only on the positive factor.

Thus, disorganization factor was not found in the dimensional structure of the Wisconsin Schizotypy Scales when exploratory factorial analyses were used and to our knowledge no studies have used confirmatory factor analyses on these scales including the Cognitive Slippage scale.

It is interesting to note that two studies [21,22] university or college samples that used CFA on several subscales of the SPQ as well as the SanS, PerAb, MIS and CSS reported a three-factor structure (negative, positive, disorganized). Social anhedonia scale loaded on the negative factor; Magical ideation and perceptual aberration scales loaded on the positive factor and the Cognitive slippage scale loaded on the disorganized factor.

However, as underlined by Kerns and Becker [23] “few studies have examined the nature of disorganized schizotypy” and only two studies have examined the relationships between disorganized schizotypy and emotional traits.

In the first study ([21], disorganized schizotypy, measured by the cognitive slippage scale, was found to be associated with increased emotional ambivalence. Ambivalence, defined as reporting simultaneously conflicting emotions in relation to a particular subject, is considered to be one of the main traits in schizophrenia [24] and schizotypy [2]. In the study by Kerns [21], disorganized but not positive schizotypy was associated with emotional confusion, mainly measured by ambivalence, and increased emotionality. In contrast, negative schizotypy including social anhedonia, was associated with increased emotional confusion but decreased emotionality. Moreover, disorganized schizotypy was associated with ambivalence even after removing the variance shared with anhedonia.

In the second study [23], subjects with disorganized schizotypy significantly differed from control participants in terms of emotional ambivalence measured by the Schizotypal Ambivalence Scale [25].

To the best of our knowledge, the relationship between disorganized schizotypy and anhedonia has not been previously investigated. Several studies in subjects presenting schizotypal features have indicated that the later onset of psychosis is linked with cognitive disorganization and anhedonia [26] and a recent follow-up study in 122 non-psychotic subjects investigated the relationship between dimension scores and transition to psychosis during the following 24 months. Scores on the negative (including anhedonia, alogia and disorganized behavior) and disorganization/cognitive dimensions were significantly associated with transition to psychosis [27].

Moreover, cognitive disorganization in schizotypy is associated with deterioration in visual backward masking [28]. Several studies have reported that the shine-through visual backward masking paradigm meets the characteristics of an endophenotype for schizophrenia [28].

However, recent research indicates that anhedonia is not a distinct entity, but can be divided into two distinct components, namely consummatory (or liking) and anticipatory (or wanting) deficit of pleasure (see review in Gard et al, [29]). Consummatory pleasure refers to the “in the moment” pleasure experienced by the subject directly engaged in an enjoyable activity, whereas anticipatory pleasure refers to the experience of pleasure related to future activities.

For Gard et al [30] the negative symptoms of schizophrenia could be related to deficits in anticipatory anhedonia. This association between anticipatory anhedonia and negative symptoms has been found also in healthy subjects (Engel et al, [31]). In subjects presenting negative schizoptypy, defined by elevated social anhedonia, high anticipatory and consummatory anhedonias have been reported comparatively to controls (Martin et al, [32]; Gooding & Pflum, [33]).

The present study was therefore designed to investigate the relationship between anhedonia and cognitive disorganization in non-clinical subjects by taking into account firstly the distinction between anticipatory and consummatory anhedonia and secondly emotional ambivalence.

Two different approaches were used. Firstly, dimensional analysis using the Cognitive Slippage scale, schizotypal ambivalence scale and two rating scales measuring anticipatory and consummatory pleasure, was conducted in a large sample of university students. Confirmatory factorial analyses tested the adequacy of various models of disorganized and negative schizotypy. Secondly, categorical analysis was performed using well-defined schizotypy (disorganized or negative) groups and non-schizotypic group based on comparisons on the ambivalence and anhedonia rating scales. Two different samples were examined; the second sample was designed to explore potential replication of the results observed in the first sample.

Given previous studies, we hypothesized firstly that disorganized schizotypy was characterized by ambivalence although negative schizopypy was characterized by anticipatory and consummatory anhedonia and ambivalence.

## Methods and results

Two different samples of non-clinical subjects were studied.

### First study

#### Participants

The sample included 64 male and 336 female university students enrolled in psychology courses at the University of Picardie. The mean age of the sample was 22.71 years (SD = 5.13). The subjects were informed about the confidentiality of their responses and gave their written informed consent to participate. The subjects were instructed to complete the questionnaires during a classroom period. This research was approved by the ethics committee of the Pinel hospital.

#### Material and procedures

Participants filled in the Cognitive Slippage Scale (CSS, Miers & Raulin, [9]), the Schizotypal Ambivalence Scale (SAS, Raulin, [10]) and the Temporal experience pleasure scale (TEPS, Gard et al, [29]). The CSS and SAS evaluate cognitive and emotional disorganization, respectively, and the TEPS measures the consummatory and anticipatory trait of pleasure. The CSS comprises 35 items related to the ability to keep track of one’s thoughts with reporting of speech deficits and/or confused thinking (Miers & Raulin, [9]). Satisfactory reliability and validity have been found in various samples, particularly in a sample of college students (Miers & Raulin, [9]). The SAS is a 19-item revision of the Intense ambivalence scale, which was designed to identify ambivalence, described by Meehl as characteristic of schizotypy and schizophrenia (Raulin, [10]; Kwapil et al, [25]). Satisfactory internal consistency and reliability in different samples of college students or young adults have been reported (Raulin, [10]; Kwapil et al, [25]). We used the French versions of the CSS and SAS that present satisfactory psychometric properties (Yon et al, [34]). In the present study, the values of the Cronbach alpha coefficient were .95 and .88 for the CSS and SAS, respectively. Moreover, the total scores (mean, SD) of the CSS and SAS were respectively 9.35 (SD = 6.74) and 7.66 (SD = 3.84). The 18-item Temporal Experience of Pleasure Scale (TEPS, Gard et al, [29]) comprises two subscales rating consummatory (TEPS-CONS) and anticipatory (TEPS-ANT) pleasures, respectively. TEPS measures trait hedonic capacity and is an indirect measure of anhedonia. Levels of anhedonia were inversely related to the scores of these subscales that can be considered as inverse measures of negative schizotypy. We used the French version of the TEPS that presents satisfactory psychometric properties (Loas et al, [35]). In the present study, the values of the Cronbach alpha coefficient was .66 for the TEPS.

Two methods were used to study the relationship between disorganization and anhedonia.

Firstly, the dimensionality of schizotypy, rated by the CSS, SAS and TEPS, was studied. Confirmatory factorial analysis (CFA) studying various factor models was performed. We used the SEPATH program of the STATISTICA software version 7.1 (Statsoft, [36]). CFA were performed using the variance-covariance matrix with the maximum-likelihood test. No correlation was allowed among the different factors. Several goodness of fit indices were used: goodness of fit index (GFI), Adjusted GFI, Normed Fit Index (NFI), Comparative Fit Index (CFI), Root Mean Square Error of Approximation (RMSEA) and the chi-square statistic. Adequate fit of the model to the data is indicated by fit indices higher than 0.95 and RMSEA less than 0.05, and non-significant chi-square statistics (Browne et al, [37]). Chi-square difference test was used to compare the various models.

Eight different models were tested to examine the factor structure. The first model included a single general factor of schizotypy. The second model included a disorganized schizotypy factor with loadings from the CSS and SAS and a negative schizotypy factor with loadings from the TEPS-ANT and TEPS-CONS. The third model was the same as the second model, except that the SAS was allowed to load onto the negative schizotypy factor. The fourth model was the same as the second model, except that the TEPS-CONS was allowed to load onto the disorganized schizotypy factor. The fifth model was the same as the fourth model, except that the SAS was allowed to load onto the negative schizotypy factor. The sixth model was the same as the second model, except that the TEPS-ANT was allowed to load onto the disorganized schizotypy factor. The seventh model was the same as the sixth model, except that the SAS was allowed to load onto the negative schizotypy factor. The eighth model was the same as the second model, except that the TEPS-ANT and TEPS-CONS were allowed to load onto the disorganized schizotypy factor.

A categorical approach was then used. Subjects with scores higher than 1.5 standard deviations above the mean on the CSS for the overall sample were included in the disorganized schizotypy group. Subjects with scores lower than .5 standard deviation above the mean on the CSS constituted the control group. These two subgroups were then compared in terms of SAS, TEPS-ANT, TEPS-CONS, age and gender by Analysis of variance or chi-square analysis. A Bonferroni correction was used (p < .05/5 = .01) to rectify the level of p.

For the missing values mean substitution was used.

#### Results

##### Dimensional analysis

Three models (5, 6, 8) provided adequate fit for the data (see Table 1): The fifth model, that included a disorganized factor with loadings from the CSS, SAS and TEPS-CONS, and a negative factor with loading from the TEPS-ANT, TEPS-CONS and SAS; the sixth model, that included a disorganized factor with loadings from the CSS, SAS and TEPS-ANT, and a negative factor with loading from the TEPS-ANT and TEPS-CONS; the eighth model, that included a disorganized factor with loadings from the CSS, SAS, TEPS-ANT and TEPS-CONS, and a negative factor with loading from the TEPS-ANT and TEPS-CONS. The more parsimonious sixth model was compared to the other two models. No significant changes were observed on chi-square test and degrees of freedom (see Table 1). Thus, the sixth model was therefore adopted. The Pearson’s correlation coefficient between total scores of the different rating scales were given in Table 2.

**Table 1:** **Goodness-of-fit indices for the theoretical models (Study 1)**

**Model**	***GFI***	***AGFI***	***NFI***	***CFI***	***RMSEA***	***x*** ^**2**^ **(** ***df*** **)**	***p***	**Δχ2 (Δ** ***df*** **)**	***p***
One-Factor	.95	.76	.82	.83	.218	42.14 (2)	.001		
2-Factor^a^	.99	.97	.95	.97	.065	10.81 (4)	.028		
2-Factor^b^	.99	.95	.97	.97	.087	8.22 (2)	.016		
2- Factor^c^	.99	.97	.98	.99	.061	5 (2)	.082		
**2- Factor** ^**d**^	1	.98	.99	1	.036	1.54 (1)	.21	1.75 (1)	ns
**2- Factor** ^**e**^	1	.98	.99	.99	.040	3.29 (2)	.19		
2- Factor^f^	1	.96	.99	.99	.074	3.17 (1)	.075		
**2- Factor** ^**g**^	1	.99	1	1	.000	.56 (1)	.46	2.73 (1)	ns

*Note*: Goodness of Fit Index (GFI); Adjusted GFI (AGFI); Normed Fit Index (NFI); Comparative Fit Index (CFI); Root Mean Square Error of Approximation (RMSEA); ns: not significant. In bold the models having adequate fit for the data.

^a^Disorganized schizotypy factor (with loadings from the Cognitive Slippage (CSS) and Schizotypal ambivalence scale (SAS)) and Negative schizotypy factor (with loadings from the anticipatory (TEPS-ANT) and consummatory (TEPS-CONS) subscales of the Temporal Experience of Pleasure Scale (TEPS)).

^b^Disorganized schizotypy factor (with loadings from the Cognitive Slippage (CSS) and Schizotypal ambivalence scale (SAS)) and Negative schizotypy factor (with loadings from the anticipatory (TEPS-ANT) and consummatory (TEPS-CONS) subscales of the Temporal Experience of Pleasure Scale (TEPS) and Schizotypal ambivalence scale (SAS)).

^c^Disorganized schizotypy factor (with loadings from the CSS, SAS and TEPS-CONS) and Negative schizotypy factor (with loadings from the TEPS-ANT and TEPS-CONS).

^d^Disorganized schizotypy factor (with loadings from the CSS, SAS and TEPS-CONS) and Negative schizotypy factor (with loadings from the TEPS-ANT, TEPS-CONS and SAS).

^e^Disorganized schizotypy factor (with loadings from the CSS, SAS and TEPS-ANT) and Negative schizotypy factor (with loadings from the TEPS-ANT and TEPS-CONS).

^f^Disorganized schizotypy factor (with loadings from the CSS, SAS and TEPS-ANT) and Negative schizotypy factor (with loadings from the TEPS-ANT, TEPS-CONS and SAS).

^g^Disorganized schizotypy factor (with loadings from the CSS, SAS, TEPS-CONS and TEPS-ANT) and Negative schizotypy factor (with loadings from the TEPS-ANT and TEPS-CONS).

**Table 2:** **Pearson’s correlations among schizotypy measures (Study 1)**

	**CSS**	**SAS**	**TEPS-ANT**	**TEPS-CONS**
CSS	1	**.62**	.05	-.03
SAS		1	**.11**	-.08
TEPS-ANT			1	**.3**
TEPS-CONS				1

Note: Anticipatory (TEPS-ANT) and consummatory (TEPS-CONS) subscales of the Temporal Experience of Pleasure Scale (TEPS); Schizotypal ambivalence scale (SAS), Cognitive slippage scale, (CSS); In bold face: p < .05).

##### Categorical analysis

The disorganized schizotypy and control groups comprised 39 subjects and 281 subjects, respectively. The two groups did not differ in terms of gender but control subjects were significantly older than disorganized subjects. Analysis of covariance was performed using the group as independent variable, age as covariate and scores of the each of the rating scales (TEPS-ANT, TEPS-CONS, SAS) as dependent variables. The disorganized schizotypy group had significantly higher scores on SAS and significantly lower scores on TEPS-CONS (more anhedonic) than controls. No significant difference in TEPS-ANT scores was observed between the two groups (see Table 3).

**Table 3:** **Self-reports and sociodemographic variables for disorganized schizotypy and control groups (Study 1)**

	**Disorganized group**	**Control group**		
	**N = 39**	**N = 281**	**p**	**ES**
Gender^a^	6 (16)	45 (16)	1	
Age^b^	20.68 (2.52)	22.96 (5.18)	.008*	
TEPS-ANT^b^	45.18 (5.50)	44.77 (5.82)	.026	.023
TEPS-CONS^b^	33.67 (5.74)	35.57 (5.58)	.0011*	.043
SAS^b^	12.21 (3.33)	6.57 (3.42)	.0001*	.25

(^a^Number (and percent) male, ^b^mean (and standard deviation), *p < .01: Bonferroni correction, Analysis of variance for age and Analysis of covariance for the three scales scores with age as covariate); anticipatory (TEPS-ANT) and consummatory (TEPS-CONS) subscales of the Temporal Experience of Pleasure Scale (TEPS); Schizotypal ambivalence scale (SAS), ES: effect size using eta square).

### Replication study

The second part of the study was designed to replicate the results of the first part of the study on a different sample.

This study was performed on the sample of a published study exploring the relationship between subjective symptoms and schizotypy in a sample of university students (Yon et al, [20]). In this study, the subjects filled in the revised Social Anhedonia Scale (RSAnS, Chapman et al, [5]; Eckblad et al, [38]), the revised Physical Anhedonia Scale (RPAS, Chapman et al, [5]; Chapman & Chapman, [39]), the CSS and the SAS. The values of the Cronbach alpha coefficients were respectively .81, .79, .89, .84.

#### Participants

The sample included 399 university students (366 women, 33 men) enrolled either in introductory psychology or nursing courses at the University of Picardie in Amiens, France. The mean age of the subjects was 24.03 years (SD = 7.17). The subjects were instructed to complete the questionnaires during a classroom period. The subjects were informed about the confidentiality of their responses and gave their written informed consent to participate. This research was approved by the local ethics committee.

#### Material and procedures

Several studies in non-clinical or clinical subjects have demonstrated significant correlations between the TEPS-ANT and the RSAnS and RPAS, whereas the TEPS-CONS was significantly correlated only with the RPAS (Gard et al, [29,30]; Loas et al, [35]). These results suggested that the RSAnS could be a measure of anticipatory anhedonia whereas RPAS could be a measure of anticipatory and consummatory anhedonia.

In the light of these findings, we extracted two subscales from the RPAS measuring consummatory anhedonia (RPAS-CONS) and anticipatory anhedonia (RPAS-ANT). Using the healthy sub-sample of the validation sample of the French version of the TEPS we had examined the correlations between the PAS items and the total scores of the TEPS-ANT or TEPS-CONS. 16 items of the PAS had high correlations with the TEPS-CONS and low correlations with the TEPS-ANT. These 16 items constituted the PAS-CONS subscale. 10 items of the PAS had high correlations with the TEPS-ANT and low correlations with the TEPS-CONS. These 10 items constituted the PAS-ANT subscale. The PAS-CONS and PAS-ANT presented significant correlations with the corresponding TEPS subscales using the validation sample of the French version of the TEPS (Loas et al, [35]).

The CSS, SAS, RPAS-ANT and RPAS-CONS were therefore used for this analysis. The values of the Cronbach alpha coefficients were .58 and .75 for the RPAS-ANT and RPAS-CONS, respectively. The total scores (mean, SD) of the CSS and SAS were respectively 6.55 (SD = 6.03) and 5.4 (SD = 4.08). For missing values mean substitution was used.

Two analyses were performed.

Firstly, dimensional analysis was performed using CFA. Eight different models were tested to examine the factor structure. No correlation was allowed among the different factors. The first model included a single general factor of schizotypy. The second model included a disorganized schizotypy factor with loadings from the CSS and SAS and a negative schizotypy factor with loadings from the RPAS-ANT and RPAS-CONS. The third model was the same as the second model, except that the SAS was allowed to load onto the negative schizotypy factor. The fourth model was the same as the second model, except that the RPAS-CONS was allowed to load onto the disorganized schizotypy factor. The fifth model was the same as the fourth model, except that the SAS was allowed to load onto the negative schizotypy factor. The sixth model was the same as the second model, except that the RPAS-ANT was allowed to load onto the disorganized schizotypy factor. The seventh model was the same as the sixth model, except that the SAS was allowed to load onto the negative schizotypy factor. The eighth model was the same as the second model, except that the RPAS-ANT and RPAS-CONS were allowed to load onto the disorganized schizotypy factor.

Secondly, a categorical approach was used. Subjects with a score higher than 1.5 standard deviation above the mean on the CSS and less than .5 standard deviation above the mean on the RSAnS for the overall sample were included in the disorganized schizotypy group. The subjects with a score higher than 1.5 standard deviations above the mean on the RSAnS and less than .5 standard deviation above the mean on the CSS for the overall sample were included in the negative schizotypy group. The subjects with a score less than .5 standard deviation above the mean on the CSS and the RSAnS constituted the control group. The disorganized, negative and control groups comprised 22, 19 and 236 subjects, respectively. Analysis of variance or chi-square tests were then used to compare these three subgroups in terms of SAS, RPAS-ANT, RPAS-CONS, age and gender. A Bonferroni correction was used (p < .01) to rectify the level of p.

#### Results

##### Dimensional analysis

Two models provided adequate fit for the data (see Table 4): The sixth model that included the disorganized factor with loadings from the CSS, SAS and RPAS-ANT, and a negative factor with loadings from the RPAS-ANT and RPAS-CONS, and the seventh model that included the disorganized factor with loadings from the CSS, SAS and RPAS-ANT, and a negative factor with loadings from the RPAS-ANT, RPAS-CONS and SAS provided adequate fit. The change in chi-square and degree of freedom was not significant between the two models. The more parsimonious sixth model was therefore adopted (see Table 4).

**Table 4:** **Goodness-of-fit indices for the theoretical models (Replication study)**

**Model**	***GFI***	***AGFI***	***NFI***	***CFI***	***RMSEA***	***χ*** **2 (** ***df*** **)**	***p***	**Δχ2 (Δ** ***df*** **)**	***p***
One-Factor	.95	.73	.84	.84	.234	46.98 (2)	.001		
2-Factor^a^	.98	.94	.93	.95	.097	18.97 (4)	.001		
2-Factor^b^	.98	.89	.94	.94	.144	18.3 (2)	.001		
2- Factor^c^	.98	.92	.96	.96	.11	12.15 (2)	.001		
2- Factor^d^	.99	.85	.96	.96	.16	11.5 (1)	.001		
**2- Factor** ^**e**^	1	.98	.99	1	.027	2.59 (2)	.27		
2- Factor^f^	1	.97	.99	.99	.057	2.25 (1)	.13		
**2- Factor** ^**g**^	1	.99	1	1	.000	.74 (1)	.39	1.85 (1)	ns

*Note*: Goodness of Fit Index (GFI); Adjusted GFI (AGFI); Normed Fit Index (NFI); Comparative Fit Index (CFI); Root Mean Square Error of Approximation (RMSEA); ns: no significant. In bold the models having adequate fit for the data.

^a^Disorganized schizotypy factor (with loadings from the Cognitive Slippage (CSS) and Schizotypal ambivalence scale (SAS)) and Negative schizotypy factor (with loadings from the anticipatory (RPAS-ANT) and consummatory (RPAS-CONS) subscales of the Revised Physical Anhedonia Scale (RPAS)).

^b^Disorganized schizotypy factor (with loadings from the CSS, and SAS) and Negative schizotypy factor (with loadings from the RPAS-ANT, RPAS-CONS and SAS).

^c^Disorganized schizotypy factor (with loadings from the CSS, SAS and RPAS-CONS) and Negative schizotypy factor (with loadings from the RPAS-ANT and RPAS-CONS).

^d^Disorganized schizotypy factor (with loadings from the CSS, SAS, and RPAS-CONS) and Negative schizotypy factor (with loadings from the RPAS-ANT, RPAS-CONS and SAS).

^e^Disorganized schizotypy factor (with loadings from the CSS, SAS and RPAS-ANT) and Negative schizotypy factor (with loadings from the RPAS-ANT and RPAS-CONS).

^f^Disorganized schizotypy factor (with loadings from the CSS, SAS, RPAS-ANT) and Negative schizotypy factor (with loadings from the RPAS-ANT, RPAS-CONS and SAS).

^g^Disorganized schizotypy factor (with loadings from the CSS, SAS, RPAS-ANT and RPAS-CONS) and Negative schizotypy factor (with loadings from the RPAS-ANT and RPAS-CONS).

The Pearson’s correlation coefficient between total scores of the different rating scales were given in Table 5.

**Table 5:** **Pearson’s correlations among schizotypy measures (Replication study)**

	**CSS**	**SAS**	**RPAS-ANT**	**RPAS-CONS**
CSS	1	**.67**	**.17**	-.07
SAS		1	**.12**	-.02
RPAS-ANT			1	**.32**
RPAS-CONS				1

Note: Anticipatory (RPAS-ANT) and consummatory (RPAS-CONS) subscales of the Revised Physical Anhedonia Scale (RPAS); Schizotypal ambivalence scale (SAS), Cognitive slippage scale, (CSS); In bold face: p < .05).

##### Categorical analysis

The three groups were not significantly different in terms of gender, but a significant difference was observed for age. Analysis of covariance was therefore performed. The three groups were significantly different in terms of RPAS-ANT, RPAS-CONS and SAS. *Post hoc* tests were not significant for RPAS-CONS. Negative schizotypy patients had significantly higher RPAS-ANT scores than control subjects. The two schizotypy groups had significantly higher SAS scores than the control group and the disorganized group had significantly higher SAS scores than the negative schizotypy group (see Table 6).

**Table 6:** **Self-reports and sociodemographic variables for disorganized schizotypy, negative schizotypy and control groups (Replication study)**

	**Disorganized group**	**Negative schizotypy**		**Control group**	
	**N = 22**	**N = 19**	**N = 236**	**p**	**ES**
Gender^a^	4 (18)	3 (16)	16 (7)	.09	
Age^b^	20.23 (2.39)	28.05 (11.09)	24.11 (6.75)	.002*	
RPAS-ANT^b^	1.95 (1.46)	2.58 (1.54)	1.32 (1.26)	0001*	.079
RPAS-CONS^b^	5.45 (2.79)	5.63 (2.54)	5.74 (2.46)	**.**0003*	.067
SAS^b^	11.36 (3.44)	6.89 (2.85)	3.87 (3.03)	.0001*	.038

(^a^Number (and percent) male, ^b^mean (and standard deviation) *p < .01: Bonferroni correction; Analysis of variance for age and Analysis of covariance for the three scales scores with age as covariate anticipatory (RPAS-ANT) and consummatory (RPAS-CONS) subscales of the Revised Physical Anhedonia Scale (RPAS); Schizotypal ambivalence scale (SAS)). *Post hoc* tests: RPAS-ANT: Negative schizotypy group > Control group (p = .0002); SAS: Disorganized schizotypy group > Control group (p = .00012). Disorganized schizotypy group > Negative schizotypy group (p = .00014): Negative schizotypy group > Control group (p = .00001). ES: effect size using eta square.

## Discussion

### Dimensional analysis

This study, using CFA in two different samples of university students, tested different models of disorganized and negative schizotypy in order to investigate the relationships between these two dimensions of schizotypy with anticipatory, consummatory anhedonia and schizotypal ambivalence.

The disorganization dimension of schizotypy is characterized by ambivalence and anticipatory anhedonia, as rated by the Schizotypal ambivalence scale and the anticipatory subscale of the TEPS, respectively. This result was found in two different samples of university students using two different anticipatory anhedonia scales. Two previous studies, using CFA, comprising 261 and 381 college students, respectively (Kerns, [21]; Cicero & Kerns, [22]), investigated the three-dimensional model of schizotypy (positive, disorganized, negative). The three dimensions were measured by several questionnaires, notably the Perceptual Aberration and Magical ideation scales for the positive component, the cognitive slippage scale and the odd beliefs subscale of the Schizotypal Personality Questionnaire for the disorganized component and the RSAnS for the negative component. The authors (Kerns, [21]; Cicero & Kerns, [22]) tested the three-dimensional model of schizotypy (positive, disorganized, negative) as well as a two-dimensional model with a positive-disorganized dimension and a negative dimension and found that the three-dimensional model provided better fit than the two-dimensional model. Unfortunately, the authors did not examine other three-dimensional models in which the RSAnS was also allowed to load onto the disorganized dimension and in which the SAS was allowed to load onto the negative dimension.

The authors (Cicero & Kerns, [22]) also tested another structural model by adding cognitive control and emotion processing variables. The three-dimension model comprising schizotypy facets (positive, disorganized, negative) predicted cognitive control and emotion traits when the three facets were included as predictors. Emotion traits were explored using several rating scales measuring emotionality and emotional confusion. Emotional confusion was defined by the SAS and the clarity of emotions subscale of the trait meta-mood scale. Disorganized schizotypy was associated with increased emotionality and increased emotional confusion, although negative schizotypy was associated with decreased emotionality and increased confusion. Schizotypal ambivalence, one of the two measures of emotional confusion, characterized disorganized and negative schizotypy.

In the two studies by Kerns (Kern, [21]; Cicero & Kerns, [22]), dimensional analyses of schizotypy therefore found a disorganized dimension characterized by cognitive slippage and a negative dimension characterized by social anhedonia and also showed that schizotypal ambivalence was related to the disorganized and negative schizotypy dimensions. Unfortunately, the authors did not test alternative dimensional models of schizotypy, in which social anhedonia or schizotypal ambivalence loaded onto both the disorganized and negative schizotypy dimensions.

The negative dimension of schizotypy was characterized by anticipatory and consummatory anhedonias. To the best of our knowledge, no previous study has investigated the dimensionality of the negative dimension of schizotypy using the TEPS. Previous studies (Lewandowski et al, [40]; Kwapil et al, [16]) using CFA have tested the dimensionality of schizotypy using several Chapman psychosis proneness scales. Using the Perceptual Aberration, Magical ideation and the revised Social (RSAnS) and Physical anhedonia (RPAS) scales in a large sample of healthy subjects, Kwapil et al [16] proposed a two-dimensional model. The perceptual aberration and magical ideation scales loaded onto the positive dimension, whereas the revised physical and social anhedonia scales loaded onto the negative dimension. Moreover, the revised social anhedonia scale also loaded onto the positive dimension. Similar results were reported by Lewandowski et al [40]. Unfortunately, the three-dimensional model of schizotypy using the Chapman psychosis proneness scales and the CSS has not been studied by CFA.

### Categorical analysis

Schizotypal ambivalence characterized disorganized schizotypy groups compared to normal or negative schizotypy groups defined by high levels of social anhedonia. These results were observed in two different samples of university students. Moreover, categorical analysis confirmed the results of dimensional analysis showing that schizotypal ambivalence characterized disorganized schizotypy.

One study (Kerns & Becker, [23]) has reported significantly higher scores on the SAS between disorganized schizotypy subjects defined by high scores on the CSS and normal subjects with low scores on the CSS. High levels of schizotypal ambivalence characterized negative schizotypy compared to control, confirming that schizotypal ambivalence is a common characteristic of negative schizotypy, defined by social anhedonia and disorganized schizotypy. Two studies have previously reported similar associations (Kerns, [21]; Cicero & Kerns, [22]). In the present study, categorical analysis suggested that schizotypal ambivalence can be used to discriminate negative and disorganized schizotypy from non-schizotypic subjects and that higher levels of schizotypal ambivalence distinguished disorganized schizotypy from negative schizotypy.

High levels of anticipatory anhedonia are characteristic of negative schizotypy. In the second study, significantly higher RPAS-ANT scores were found in negative schizotypy subjects compared to controls. Two studies have compared negative schizotypy subjects, defined by high scores on the revised social anhedonia scale, and controls, on the basis of TEPS-ANT and TEPS-CONS scores. Martin et al [32] found significantly lower scores (higher anhedonia) on TEPS-ANT and TEPS-CONS for negative schizotypy subjects compared to controls. Goodin and Pflum [33] reported similar results. In the present study, no significant difference for RPAS-CONS scores was observed between negative schizotypy subjects and controls. Several studies have reported significant correlations between the RSAnS and the TEPS-ANT, although the RPAS was significantly correlated with the TEPS-ANT and TEPS-CONS (Gard et al, [29,30]; Loas et al, [35]). Social anhedonic subjects may therefore present only higher anticipatory anhedonia, and not consummatory anhedonia as rated by the RPAS-ANT and RPAS-CONS, respectively.

The first part of this study demonstrated high levels of consummatory anhedonia in disorganized schizotypy subjects compared to normal subjects. This result, using the TEPS-CONS, was not confirmed in the second part of the study using the RPAS-CONS. As demonstrated by Strauss and Gold [41], a considerable variability in control TEPS-ANT and TEPS-CONS scores has been observed in the various studies on schizotypy (Gooding and Pflum, [33]; Martin et al, [32]). This variability could explain the equivocal results observed in our study.

### Limitations

This study presents a number of limitations. Firstly, CFA was performed in two groups of university students of both genders, but the results of these analyses must be confirmed separately in each gender and also in samples representative of the general population. In university students, one study reported that the factor structure of the Wisconsin schizotypy scales was found to be invariant across participants’ gender and age (Fonseca-Pedrero et al, [15]). No infrequency scale was used to screen out subjects who responded in a random or “fake-bad” manner. Secondly, the different samples used for categorical analysis in the two studies were not matched for gender and age. In view of the marked variability of TEPS-ANT and TEPS-CONS scores observed in healthy controls, our results need to be replicated using disorganized, negative schizotypy and control subjects matched for gender and age. Thirdly, the psychometric properties of the subscales extracted from the RPAS must be verified in other samples. Fourthly, the study has used only self-report measures and the results must be confirmed using defined of the schizotypy made by interviewed based measures. Fifthly, the results of the present cross-sectional study must be confirmed by prospective studies allowing to verify the stability of the significant associations.

## Conclusion

Despite these limitations, our study is the only one to examine the relationship between anhedonia and disorganized schizotypy and to take into account ambivalence as well as the distinction between consummatory and anticipatory pleasure. The results suggested firstly that ambivalence was not specific of disorganized schizotypy and secondly that anticipatory anhedonia was not specific of negative schizotypy. Thirdly, consummatory anhedonia could characterize only negative schizotypy.

## Abbreviations

CFA: Confirmatory Factorial Analysis
CSS: Cognitive slippage scale
INC: Impulsive nonconformity scale
MIS: Magical ideation scale
PerAb: Perceptual aberration scale
TEPS: Temporal experience pleasure scale
TEPS-CONS: Consummatory subscale of the TEPS
TEPS-ANT: Anticipatory subscale of the TEPS
RPAS: Revised physical anhedonia scale
RSAnS: Revised social anhedonia scale
SAS: Schizotypal ambivalence scale
SF: Social fear scale
SPQ: Schizotypal Personality Questionnaire
